# Antimicrobial ceragenins inhibit biofilms and affect mammalian cell viability and migration *in vitro*

**DOI:** 10.1002/2211-5463.12235

**Published:** 2017-05-22

**Authors:** Melissa A. Olekson, Tao You, Paul B. Savage, Kai P. Leung

**Affiliations:** ^1^ Dental and Craniofacial Trauma Research & Tissue Regeneration DirectorateUnited States Army Institute of Surgical ResearchJBSAFort Sam HoustonTXUSA; ^2^ Department of Chemistry and BiochemistryBrigham Young UniversityProvoUTUSA

**Keywords:** antimicrobials, biofilms, wound healing

## Abstract

The healing of burn wounds is often hampered by bacterial infection and the formation of biofilms. Antimicrobial peptides (AMPs) are effective in promoting wound healing, but are susceptible to degradation. We have tested the ability of ceragenins (CSAs), mimics of antimicrobial peptides, to mitigate preformed biofilms and stimulate wound healing *in vitro*. Potent CSAs (MICs < 10 μg·mL^−1^) were tested against biofilms formed from a mixture of *Pseudomonas aeruginosa* and *Staphylococcus aureus* grown for 22 h and subjected to 20 h treatment. Many CSAs showed more potent anti‐biofilm activity than the endogenous AMP LL‐37, and CSA‐13 and CSA‐90 decreased the amount of biofilm matrix substances detected by SYPRO Ruby stain. Effects on mammalian cells were measured by viability, migration, and tube formation assays *in vitro*. Although CSAs were toxic to immortalized human keratinocytes (HaCaTs) at higher concentrations (>10 μg·mL^−1^), lower concentrations of CSA‐13 and CSA‐192 stimulated cell migration. CSA‐13, CSA‐90, and CSA‐142 also stimulated tube formation in an *in vitro* angiogenesis model. An inhibitor of vascular endothelial growth factor receptor 2 (VEGFR2) blocked tube formation stimulated by CSA‐13, suggesting that CSA‐13 signals through this receptor. Ceragenins display anti‐biofilm activity and stimulate migration and tube formation *in vitro*. This work suggests that ceragenins have the potential to be both topical antimicrobials and wound‐healing adjunct therapeutics.

**Abbreviations**
AMP: antimicrobial peptide
BHI++: brain heart infusion media with 2% NaCl and 1% glucose
CFU: colony forming unit
CSA: ceragenin
CV: coefficient of variance
DMEM: Dulbecco's modified Eagle's medium
EBM: endothelial basal medium
ECIS: electric cell‐substrate impedance sensing
EGF: epidermal growth factor
FBS: fetal bovine serum
HaCaT: immortalized human keratinocytes
HUVEC: human umbilical vein endothelial cells
LPS: lipopolysaccharide
MBC: minimum bactericidal concentration
MHB: Mueller Hinton broth
MTT: 3‐[4,5‐dimethylthiazol‐2‐yl]‐2,5‐diphenyl tetrazolium bromide
OD: optical density
P/S: penicillin/streptomycin
PBS: phosphate buffered saline
SEM: scanning electron microscopy
TGFβ1: transforming growth factor β1
TPMT: transmission photomultiplier tube
TSB: tryptic soy broth
VEGFR2: vascular endothelial growth factor receptor 2
VEGF: vascular endothelial growth factor

Burn wounds are devastating injuries that incur a mortality rate of 5–10% for both civilian and military populations 1. During the course of healing, several phases occur: (a) an initial inflammatory phase where neutrophils and, later, macrophages clear infection and debris; (b) a secondary proliferation phase involving fibroblasts that populate the granulation tissue, keratinocytes that begin to close the wound, and endothelial cells that undergo angiogenesis to form the new vascular network; and (c) a final remodeling phase where collagen reorganizes and myofibroblasts contract the wound 2. In burn injuries, the leading cause of death is infection 1, 3. Wound bacterial colonization is commonly polymicrobial and can lead to the formation of biofilm, a phenotype of aggregated bacteria and extracellular polymeric substances; biofilms have increased virulence and resistance to treatment that can worsen wound outcomes 4, 5. Of great clinical concern are the ESKAPE group of pathogens, which encompass drug‐resistant Gram‐negative and Gram‐positive species that lead to the majority of hospital acquired infections and related deaths 6. Two of the ESKAPE pathogens are *Pseudomonas aeruginosa* and *Staphylococcus aureus*, the most common Gram‐negative and Gram‐positive organisms, respectively, in burn wounds 7. With fewer strains responding to current antibiotic treatments, the need for novel antimicrobials is paramount.

Traditional antimicrobial topical treatments used for burn wounds are silver‐containing antimicrobials such as silver sulfadiazine. These, together with a sulfonamide‐type medication, such as mafenide acetate, are the standard of care of infected burn wounds in some treatment centers 2. The drawbacks associated with these agents are that they can be detrimental to the patient, causing pain and impaired healing 8, 9, 10. In addition, silver sulfadiazine may cause increased scarring 11. Therefore, there has been an effort to shift from traditional treatments to alternatives that have lessened detrimental effects on human tissue. Recent findings with antimicrobial peptides (AMPs), such as human LL‐37, suggest that they are an attractive option for providing topical anti‐biofilm activity and promotion of the wound‐healing cascade. LL‐37 provides broad‐spectrum killing of bacteria by disrupting their cell membranes. In eukaryotes, LL‐37 has been shown to decrease inflammation and induce migration and angiogenesis pathways; however, this peptide is rapidly degraded, especially at sites of infection, and is hemolytic at higher concentrations 12. While some have shown LL‐37 has anti‐biofilm activity 13, there are others that show LL‐37 lacks the ability to disrupt preformed biofilms 14. In addition, some strains can develop LL‐37 resistance mechanisms 15. An optimal therapeutic for burn wounds would mimic the beneficial effects of LL‐37, including broad‐spectrum antibacterial, anti‐inflammatory, and wound‐healing activities, while remaining stable in wounds and displaying anti‐biofilm activity.

One of the antibacterial alternatives that could have beneficial effects on the healing of infected burn wounds is a group of antimicrobial peptide‐like molecules called ceragenins. Ceragenins are cholic acid derivatives that have been modified to mimic charge characteristics and amphipathy of AMPs like LL‐37. While they exhibit broad‐spectrum antimicrobial and anti‐biofilm activity 16, 17, their mimicry of AMP structures also leads to pleiotropic effects that parallel functions induced by AMPs. Though not been previously tested in wound‐healing models, ceragenin CSA‐13 has been shown to sequester lipopolysaccharides (LPS) 18 and induce the release of IL‐8 from HaCaTs. In addition, because ceragenins are not peptides, they are resistant to protease degradation 16. As with AMPs, bacterial pathogens are less likely to generate resistance to ceragenins, such as CSA‐13, than to other antibiotics. For example, in a recent study, *S. aureus* remained fully susceptible to CSA‐13 after 30 days of serial passaging, while the minimum inhibitory concentration (MIC) of ciprofloxacin increased more than a100‐fold to over 130 μg·mL^−1^ over the same period. With *P. aeruginosa*, the MIC of CSA‐13 increased to 20 μg·mL^−1^ (10‐fold increase) under these conditions, while the MIC of colistin increased to over 200 μg·mL^−1^ (1000‐fold increase) 19.

The studies described herein compared the antibacterial potency of 14 ceragenin molecules against multidrug‐resistant Gram‐negative and Gram‐positive bacteria. Seven down‐selected agents from initial antibacterial screening displayed differences in agent‐induced anti‐biofilm activity against preformed biofilms and mammalian cytotoxicity. Select ceragenins were able to enhance would‐healing processes *in vitro*: CSA‐13 and CSA‐192 were able to stimulate HaCaT migration, and CSA‐13, CSA‐90, and CSA‐142 were able to stimulate human umbilical vein endothelial cell (HUVEC) tube formation, an *in vitro* measurement for the ability of the endothelial cells to form vessels during the proliferation phase of wound healing. CSA‐13 was unable to induce tube formation when VEGFR2 signaling was blocked, suggesting signaling through VEGFR2 in HUVECs. Our findings suggest the potential of ceragenins as wound adjunct treatments.

## Materials and methods

### Anti‐biofilm compounds, strains, and growth media

Ceragenins were prepared following previously described procedures 20, 21. LL‐37 was purchased from Anaspec (Fremont, CA, USA). Drug‐resistant *P. aeruginosa* (PAO1 – University of Washington) and *S. aureus* subsp. *aureus* (ATCC^®^ BAA‐1717™) were used in this study. Overnight bacterial cultures grown in tryptic soy broth (TSB) were diluted 1 : 20 and grown to an optical density of (OD) 0.5 at the wavelength of 600 nm. HaCaTs were cultured in full growth medium, consisting of Dulbecco's modified Eagle's medium (DMEM) supplemented with 10% v/v fetal bovine serum (FBS) and 1% v/v penicillin/streptomycin (P/S) (all reagents were purchased from ThermoFisher Scientific; Waltham, MA, USA). Primary HUVECs from a single donor were cultured for up to 15 population doublings in endothelial basal medium (EBM) supplemented with the SingleQuots kit which adds a mixture of growth factors and 2% FBS (cells and reagents purchased from Lonza, Walkersville, MD, USA). All cells were maintained at 37 °C, 5% CO_2_ in T‐75 flasks (Sarstedt, Numbrecht, Germany).

### *In vitro* susceptibility

Minimum inhibitory concentration and minimum bactericidal concentration (MBC) measurements were based on the National Committee for Clinical Laboratory Standards broth microdilution method with minor modifications as previously described 22. Briefly, bacteria were suspended in 2× Mueller Hinton broth (MHB), adjusted to an optical density (OD) of 0.05 at 600 nm and diluted 1 : 10. Test agents in sterile deionized (DI) H_2_O were serially diluted two‐fold from 2× of the highest concentration (200 μg·mL^−1^). Each well in a 96‐well plate received 100 μL test agent and 100 μL bacterial suspension distributed by a Microlab STARlet (Hamilton Robotics, Reno, NV, USA), resulting in a final bacteria concentration of 2 × 10^6^ colony‐forming units (CFU) mL^−1^. Plates were incubated overnight at 37 °C, ambient air. MIC was determined as the lowest concentration that prevented visible turbidity at 600 nm (Synergy™ HT; Biotek, Winooski, VT, USA). For viable counts, 50 μL of bacterial suspension was plated onto 5% sheep blood agar plates and incubated overnight at 37 °C, ambient air. MBC was determined as the lowest concentration that prevented overnight growth. Results presented are representative of at least three independent experiments.

### Anti‐biofilm activity

Bacteria were adjusted to an OD of 0.05 at 600 nm in phosphate‐buffered saline (PBS). A 1 : 1 mixture of *P. aeruginosa* and *S. aureus* (400 μL total volume) were incubated together on each 5 mm borosilicate glass disc (Ace Glass, Vineland, NJ, USA) with one disc per well in a 48‐well plate for 2 h. After washing in PBS, 400 μL Brain Heart Infusion media with 2% NaCl and 1% Glucose (BHI++) and supplemented with 10% human plasma was added per well. The discs were incubated for 22 h at 37 °C in a shaker incubator. Each disc was rinsed and LL‐37 or CSAs were added in 400 μL BHI++. After a second incubation of 20 h, the discs were once again washed in PBS.

With the 1 : 1 plating ratio, there was no difference in the amount of each bacteria after 2 h (4.85 *Pseudomonas* vs 4.48 *S. aureus* mean log CFU/disc). After 22 h and 42 h of incubation time, *S. aureus* dominates the culture, with 6.26 *Pseudomonas* vs 7.69 *S. aureus* mean log CFU/disc and 5.32 *Pseudomonas* vs 7.76 *S. aureus* mean log CFU/disc, respectively. The dominance of one bacterial species over the others in a mixed‐species setting is likely due to the type of culture medium used *in vitro*. We have demonstrated previously that BHI++ medium promotes the growth of *S. aureus* 23. Individually, a planktonic culture of *S. aureus* grows faster under these growth conditions than pseudomonas. Altering media nutrients can change species dominance, but no recipe gives an equal amount of the two species; in fact, even in animal models, species cannot be maintained at a perfect 1 : 1 ratio 24. To mimic some of the *in vivo* environment, BHI++ medium was supplemented with human plasma in the test mixed‐species biofilms.

For counting cells, the discs were placed in 1 mL PBS. Bacteria were detached by sonication (Microson XL; Misonix, Farmingdale, NY, USA), plated (WASP II; Don Whitley Scientific, West Yorkshire, UK) on 5% sheep blood agar (and in some cases on selective media for *Pseudomonas* and *Staphylococcus*, respectively), and incubated overnight at 37 °C, ambient air for bacterial enumeration with a counter (Protocol3; Synbiosis, Frederick, MD, USA). Bacteria counts for treated biofilms were graphed as a reduction from the control media‐treated cells. A negative reduction means that there were more cells in the treated group than control group. Data presented as box and whisker plot for *N* = 3 experiments.

### Scanning electron microscopy

For scanning electron microscopy (SEM), glass discs were precleaned with acetone, ethanol, and water before sterilization. Biofilms were grown as stated above and rinsed by water before being fixed by 2.5% phosphate‐buffered glutaraldehyde for 30 min at 4 °C as previously described 25. The fixed samples were dehydrated in a graded series of cold ethanol/water mixture (increasing from 10%, 20%, 30%, 50%, 70%, 80%, 90%, 95% to 100% ethanol) for 10 min each. Critical point dryer (Leica EM CPD300; Leica Microsystems, Buffalo Grove, IL, USA) was used to dehydrate the samples after which the samples were coated with a gold/palladium target using a high vacuum sputter coater (Leica ACE600; Leica Microsystems). The biofilm discs were observed with a SIGMA VP40 field emission scanning electron microscope (Carl Zeiss, Inc. Oberkochen, Germany) in high vacuum mode at 2 kV. Results presented are representative of at least three independent experiments. Cell diameters in SEM images were measured via imagej (NIH, Bethesda, MD, USA). For each condition, a total of 150 cells were measured across three separate experiments. Data presented as mean ± SEM. Distributions were created using bins of 0.2 μm. Coefficient of variance (CV) was calculated using the formula:

CV(%)=standard deviation(σ)/mean(μ)∗100

### Measurement of biofilm matrix substances

Bacteria were adjusted to an OD of 0.05 at 600 nm in PBS. A 1 : 1 mixture of *P. aeruginosa* and *S. aureus* (100 μL total volume) was incubated together per well in a 96‐well plate for 2 h. After washing in PBS, 100 μL BHI++ with 10% human plasma was added per well. The plate was incubated for 22 h at 37 °C in a shaker‐incubator and then rinsed twice in PBS. At this time, some wells were stained with SYPRO Ruby Biofilm Matrix Stain (ThermoFisher Scientific) and others were treated with control media, LL‐37, or CSAs in 100 μL BHI++ and incubated for an additional 20 h (42 h total culture) before washing and staining. For staining, 100 μL of SYPRO Ruby was added to each well in the dark and incubated for 30 min at room temperature. The wells were rinsed twice with DI H_2_O and resuspended in 100 μL of DI H_2_O before fluorescence was assessed at excitation: 450 nm/emission: 610 nm (Synergy™ HT, Biotek). Data presented as mean ± SEM for *N* = 4 experiments.

### HaCaT viability via MTT test

Immortalized human keratinocytes viability was estimated by the ability of the cells to enzymatically reduce 3‐[4,5‐dimethylthiazol‐2‐yl]‐2,5‐diphenyl tetrazolium bromide (MTT) to formazan (Tox1 kit; Sigma‐Aldrich, St. Louis, MO, USA). HaCaTs were plated at 25 000 cells per well in a 96‐well plate (100 μL per well) and grown to confluence for 24 h at 37 °C, 5% CO_2_. The cells were treated with LL‐37 or CSAs in full growth medium. After 24 h of additional incubation, the cell supernatant was removed and replaced with MTT (10 μL per well). After 4 h of incubation at 37 °C, 5% CO_2_, the MTT crystals were dissolved using MTT Solubilization Solution (100 μL per well). After 1 min of shaking, absorbance was read at 570 nm/corr: 690 nm (Synergy™ HT, Biotek). Percent viability was determined by normalizing absorbance to that of control cells treated only with full growth medium. Data presented as mean ± SEM for *N* = 4 experiments.

### HaCaT 2‐D wounding via electric cell‐substrate impedance sensing

The electric cell‐substrate impedance sensing (ECIS) system (Applied Biophysics, Troy, NY, USA) allows for electronic monitoring of cells on well‐arrays containing gold plated electrodes. As the cells cover the electrodes, impedance of current increases, which is measured as the output. A short pulse of a current 1000‐fold higher in magnitude causes cell death on the electrode, resulting in a 2‐D ‘wound’. The procedure was adapted from prior work 26. All experiments were performed on 96W1E+ ECIS arrays (Applied Biophysics). Prior to inoculation, the electrodes were cleaned with 10 mmol·L^−1^ l‐cysteine (pH 7.5, Sigma‐Aldrich) and rinsed twice with DI H_2_O. HaCaTs were plated at 60 000 cells per well in 200 μL of full growth medium at 37 °C, 5% CO_2_. After growing to confluence in 16–24 h, as shown by a plateau in impedance, the medium is switched to serum‐free DMEM with 1% P/S for 24 h at 37 °C, 5% CO_2_. After serum starvation, each well received a ‘wound’ of 30 s at 5000 μA, 100 μL media was removed, and 100 μL treatments of LL‐37 and CSA‐13 were added at 2 ×  concentration. Migration response of impedance recovered over time was normalized to the control cells treated with serum‐free media. Data presented as mean ± SEM for *N* = 5 experiments.

### HUVEC tube formation

Each well of a μ‐plate angiogenesis 96‐well (ibidi, Madison, WI, USA) was coated with 10 μL of growth factor‐reduced, phenol red‐free matrigel (Corning, Corning, NY, USA), and placed at 37 °C, 5% CO_2_ for 30 min. HUVECs were plated at 420 000 cells per mL in EBM supplemented only with 2% FBS. A mixture of 35 μL of cell suspension and 35 μL of LL‐37 or CSAs at 2× concentration were added to each well. Plates were incubated at 37 °C for 6 h at 5% CO_2_. For imaging, cells were labeled with Calcein AM (ThermoFisher Scientific) at 4 μmol·L^−1^ for 30 min in PBS. Tube formation for each condition was assessed from three 5× objective fields; one field per well (Zeiss Axio Vert A.1; Oberkochen, Germany). Some conditions included ZM 323881 hydrochloride (Bio‐Techne, Minneapolis, MN, USA), a selective inhibitor of VEGFR2 27. Data presented as mean ± SEM for *N* = 5 experiments.

### Intracellular calcium release

Human umbilical vein endothelial cells were plated at 400 000 cells per mL in 100 μL per well in a 96‐well plate. After overnight growth to reach confluence, the cells were switched to serum‐free media (basal EBM) for 4 h prior to testing. Fluo‐4AM (ThermoFisher Scientific) was added in basal media at 4 μmol·L^−1^ (100 μL per well) and incubated for 45 min at 37 °C. Cells were washed twice in a buffer of Hank's Balanced Saline Solution plus 3.3 mmol·L^−1^ CaCl_2_. For some cells, ZM 323881 inhibitor was pre‐incubated at 10 μmol·L^−1^ for 30 min prior to imaging. Fluorescent images were captured from three 10 ×  objective fields (one field per well, 0.45 NA) using a confocal laser scanning microscope (488 excitation from Argon ion laser, LSM 710 – Carl Zeiss). Transmitted images captured using transmission photomultiplier tube (TPMT). Baseline calcium activity images of the well were taken prior to adding the inducing agent [control, vascular endothelial growth factor (VEGF), or CSA‐13]. Ionomycin calcium salt, an ionophore, was used as the positive control. After adding, a time lapse of images was taken every 10 s for a total of 4 min. Mean fluorescence intensity and percent fluorescent area of the cell‐covered area were quantified using ImageJ and normalized to the ionomycin condition. Area under the curve for normalized data was calculated by graphpad prism (GraphPad Software, La Jolla, CA, USA). Data presented as mean ± SEM for *N* = 3 experiments.

### Statistics

Data were tested for adherence to normal distribution using goodness‐of‐fit test (JMP). Normally distributed data were tested using Student's *t*‐test or ANOVA with post‐tests noted in figure legends (graphpad prism). Data which were not normally distributed were tested with the nonparametric Kruskal–Wallis with Steel post‐test (JMP). Differences were considered to be statistically significant for *P* < 0.05.

## Results

### *In vitro* susceptibility of planktonic bacteria to ceragenin peptide mimics

**Figure 1:**
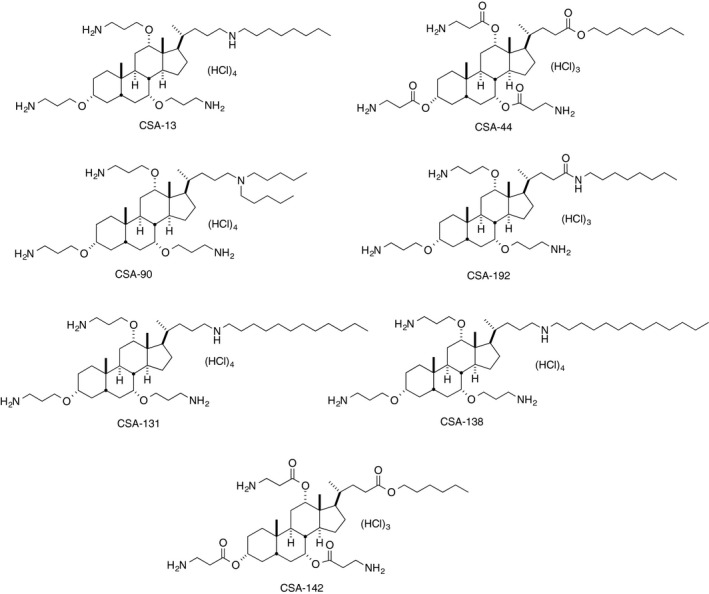
Structure of ceragenins selected for investigation.

PAO1 and ATCC^®^ BAA‐1717™ were tested for susceptibility to ceragenins, and the MICs and MBCs for ceragenins were much lower than those for LL‐37 (Table 1), with several compounds (CSA‐13, 44, 90, 192, 131, 138, and 142) having MIC/MBC values less than 10 μg·mL^−1^ for both strains. These compounds (Fig. 1), along with LL‐37 for comparison, were selected for further analysis.

**Table 1:** Minimum inhibitory concentration and MBC of LL‐37 and ceragenins to *Pseudomonas aeruginosa* and *Staphylococcus aureus*

	MIC/MBC *P. aeruginosa* (μg·mL^−1^)	MIC/MBC *S. aureus* (μg·mL^−1^)
LL‐37	>200/>200	>200/>200
CSA‐8	25/100	3.125/6.25
CSA‐11	>200/>200	50/50
CSA‐13	3.125/6.25	0.78/0.78
CSA‐25	25/50	1.56/3.25
CSA‐44	3.125/6.25	1.56/3.25
CSA‐54	50/100	6.25/25
CSA‐90	6.25/6.25	1.56/1.56
CSA‐192	3.125/6.25	0.78/1.56
CSA‐131	3.125/3.125	0.78/1.56
CSA‐134	12.5/25	0.78/3.125
CSA‐138	3.125/6.25	1.56/3.125
CSA‐142	3.125/3.125	3.125/3.125
CSA‐144	12.5/50	3.125/3.125
CSA‐145	12.5/50	3.125/3.125

### Ceragenins disrupt preformed mixed‐species biofilms

**Figure 2:**
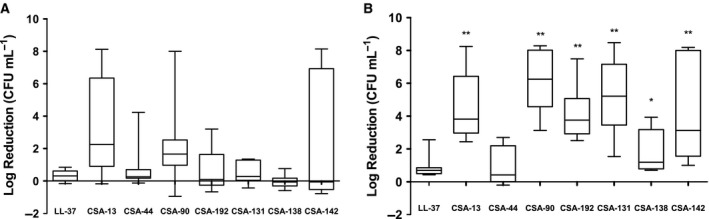
Effect of ceragenins on the CFU·mL^−1^ count of a mixed‐species *P. aeruginosa/S. aureus* biofilm after 20‐h incubation with 10 μg·mL^−1^ (A) and 100 μg·mL^−1^ (B) doses. Data points show the log reduction compared to the untreated media control and represent the median, 25th/75th percentile (boxes), and min/max (whiskers) of three independent experiments. The levels of statistical significance of the ceragenins compared to LL‐37 are as follows: **P < 0.01; *P < 0.05 (nonparametric Kruskal–Wallis with Steel post‐test).

**Figure 3:**
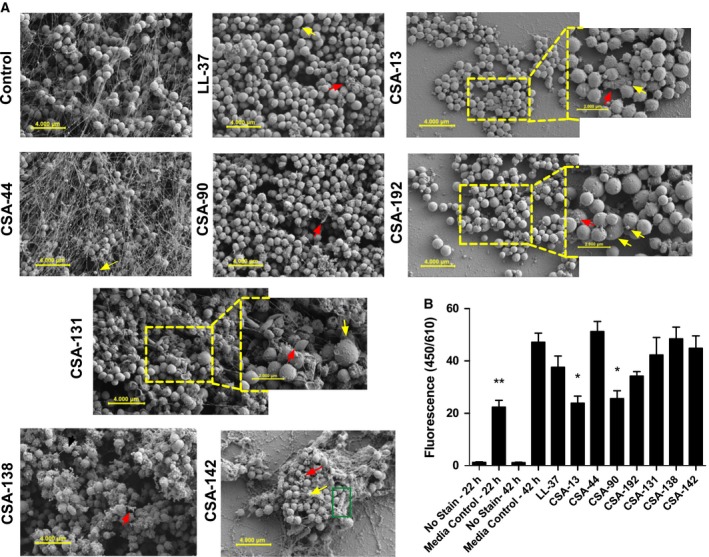
Scanning electron micrographs showing representative results of mixed‐species *P. aeruginosa/S. aureus* biofilm treated with 100 μg·mL^−1^ for 20 h (A) and effect of the same dose on SYPRO ruby biofilm matrix stain (B). Data points show the mean fluorescence (arbitrary units) of three independent experiments ± SEM. The levels of statistical significance of the treatments compared to the media control at 42 h are as follows: **P < 0.01; *P < 0.05 (ANOVA with Dunnett's post‐test).

Mixed‐species PAO1/ATCC^®^ BAA‐1717™ biofilms grown on glass discs were plated for colony counts after treatment with 10 μg·mL^−1^ (Fig. 2A) and 100 μg·mL^−1^ (Fig. 2B) of each test compound. A dose–response occurred with ceragenin treatment. Compared with LL‐37, CSA‐13, 90, 192, 131, 138, and 142 at 100 μg·mL^−1^, all significantly reduced the amount of live bacteria in the biofilm. Scanning electron microscopy was used to observe cell morphology and biofilm structure (Fig. 3A). The images presented are a selection from at least three separate experiments per treatment. The media‐treated control biofilms were ATCC^®^ BAA‐1717™ dominant and contained smooth, spherical cells with a diameter of ~ 1 μm. The presence of extracellular matrix materials was also detected. PAO1 was visible in some areas (marked with green rectangle). As detected by morphology, the same *S. aureus* dominance was seen in treated conditions, so counts and size changes were assumed to be primarily for this species. During treatment with LL‐37, some debris was visible (red arrow), and the diameter in some cells increased (yellow arrow). In contrast, treatment with CSA‐13 reduced the amount of extracellular material, induced dead cells/debris (red arrow), and induced a morphology change of decreased cell diameter (yellow arrow). CSA‐44 treatment also resulted in the appearance of smaller than normal cells (yellow arrow). Treatment with CSA‐90 showed a decrease in extracellular matrix with some debris (red arrow). The response to CSA‐192 showed areas of dead material (red arrow) and cells of different diameters (yellow arrows). CSA‐131 treatment induced what appeared to be spheroidal dead cells (red arrow) and an increase in diameter in some cells (yellow arrow). Dead cells/debris (red arrow) was also observed with the treatment of CSA‐138. CSA‐142 had area of debris (red arrow) and cells of different diameters (yellow arrow). SYPRO Ruby fluorescent staining was used to determine differences in total biofilm matrix quantity between control media and ceragenin/LL‐37‐treated biofilms (Fig. 3B). From 22 h to 42 h of biofilm growth in control media, there is a significant increase in matrix production. Compared to the media control at 42 h, CSA‐13‐ and CSA‐90‐treated cells had significantly decreased matrix substance production, while the other ceragenins and LL‐37 did not significantly decrease the amount of matrix at 42 h.

**Figure 4:**
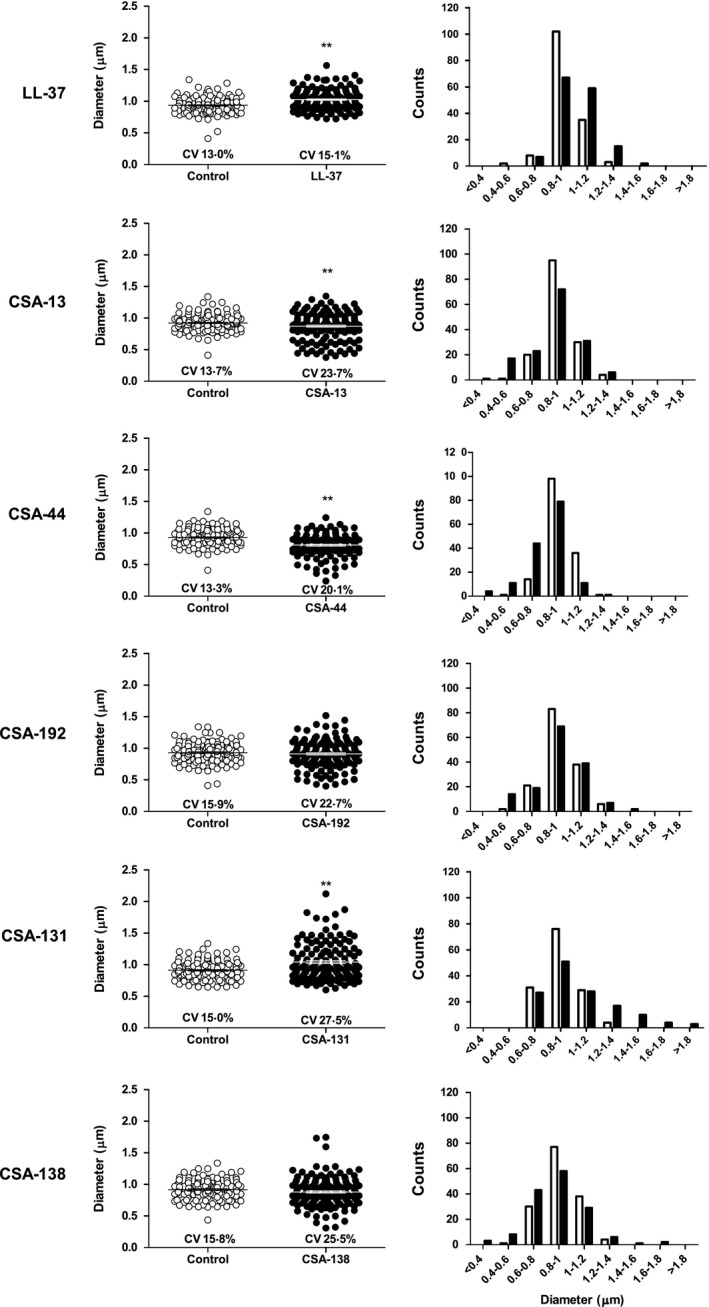
Quantification of cell diameter size and size distribution in control (open) and treated (closed) conditions from SEM images. CV, coefficient of variance. Size data points show mean diameter of three independent experiments ±SEM. The levels of statistical significance of the treatment compared to the media control are as follows: **P < 0.01 (Student's *t*‐test). Distribution data points show number of cells per 0.2 μm bin from three independent experiments.

Because many treatments affected cell size, cell diameter was measured and a distribution of diameters for each treatment was graphed (Fig. 4). We determined coefficient of variance (CV), which is a ratio of standard deviation to the mean, and which increases with heterogeneity of the population. For LL‐37, the increase in cell diameter was significant, and the shift to a population with increased cell diameter is evident from the distribution histogram. CV was similar between control and LL‐37‐treated cells. Cells treated with CSA‐13 had a significantly smaller diameter, as evident by the addition of a group of cells with a diameter of 0.4–0.6 μm and larger CV. The results for CSA‐44 were similar to those with CSA‐13; the diameter significantly decreased, the CV increased, and more cells were less than 0.8 μm as compared to control. Cells treated with CSA‐192 did not differ in mean diameter, but did have a larger CV, with more cells at the low end of the distribution. For CSA‐131, the average diameter was significantly increased as many cells had diameters greater than 1.2 μm on the distribution; the CV was nearly double that of the control. Treatment with CSA‐138 was similar to that of CSA‐192, with no change in average diameter with treatment, but there was higher CV. The CSA‐90‐ and CSA‐142‐treated cells were very similar to control media‐treated cells in mean diameter and population distribution (data not shown).

### HaCaT cytotoxicity and 2‐D wound healing

**Figure 5:**
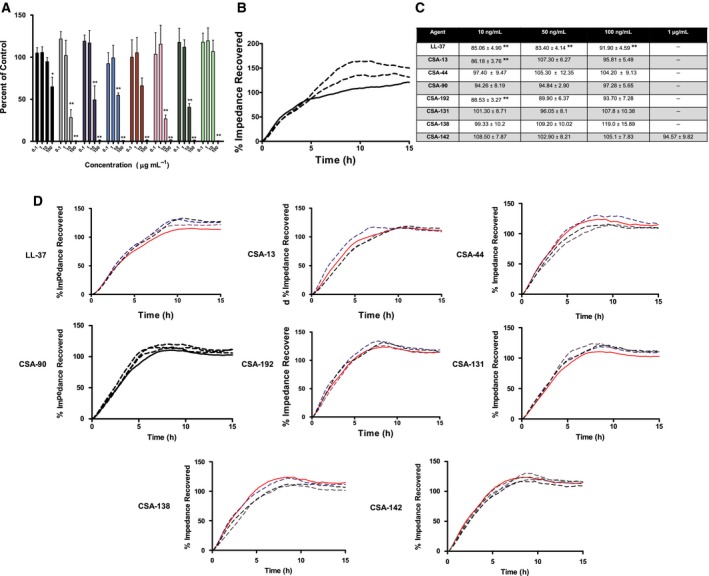
Effect of ceragenins on viability of HaCaT cells as measured via MTT assay (A). Treatments consist of LL‐37 (black), CSA‐13 (gray), CSA‐44 (dark blue), CSA‐90 (light blue), CSA‐192 (red), CSA‐131 (pink), CSA‐138 (dark green), and CSA‐142 (light green). Data points show viability as percent of control‐treated cells and are a mean of four independent experiments ± SEM. The levels of statistical significance of the treatment compared to the media control are as follows: **P < 0.01; *P < 0.05 (two‐way ANOVA with Bonferroni post‐test). Representative ECIS responses are shown for growth factors (B). Treatments consist of control media (red solid), epidermal growth factor at 5 ng·mL^−1^ (brown dash), and transforming growth factor β1 at 2 ng·mL^−1^ (yellow dash). Ceragenin effect on ECIS cell migration (C). Data in the table show time to closure as percent of control‐treated cells and are a mean of five independent experiments ± SEM. The levels of statistical significance of the treatment compared to the media control are as follows: **P < 0.01 (nonparametric Kruskal–Wallis with Steel post‐test). Representative ECIS responses are shown for test conditions (D). Compared to control (red solid), stated treatments are at doses of 10 ng·mL^−1^ (blue dash), 50 ng·mL^−1^ (purple dash), 100 ng·mL^−1^ (black dash) for all ceragenins and additionally 1 μg·mL^−1^ (green dash) for CSA‐142.

Though they are more potent, the ceragenins are also more cytotoxic to the HaCaT cell line than LL‐37 as determined by the MTT assay. Except for CSA‐192 and CSA‐142, there was a significant decrease in viability with ceragenins at 10 μg·mL^−1^; for LL‐37, the significant decrease in viability occurred at a treatment of 100 μg·mL^−1^ (Fig. 5A).

Immortalized human keratinocytes cells were used in a 2‐D measure of wound healing by ECIS. Representative runs using growth factor controls are shown in Fig. 5B. Migration stimulators such as epidermal growth factor (EGF) and transforming growth factor β1 (TGF β1) induced faster closure (less time to recover to 100% prewound impedance) than the control and ended at a higher impedance; the increase in impedance could be from growth factor‐induced morphology changes or proliferation at later time points. LL‐37 induced a significant increase in migration at all concentrations tested (10, 50, and 100 ng·mL^−1^) as noted by the shift in the curves; CSA‐13 and CSA‐192 induced a significant increase in migration at only the lowest concentration (10 ng·mL^−1^, Fig. 5C,D).

### HUVEC tube formation

**Figure 6:**
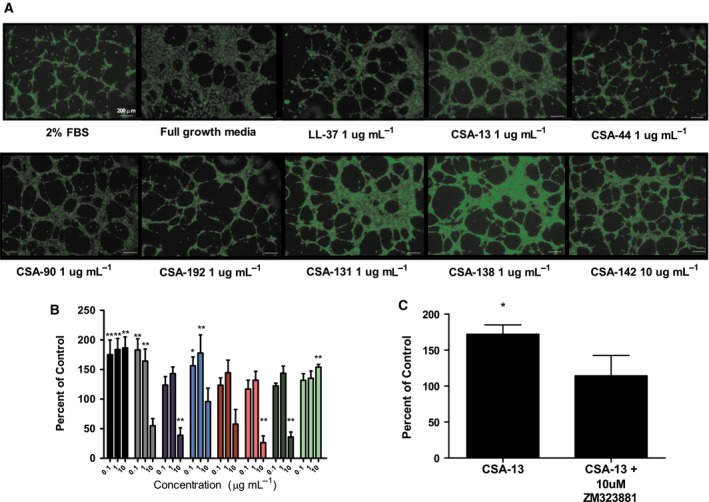
Human umbilical vein endothelial cells treated with ceragenins showed many connected loops compared to 2% FBS control where there were many breaks in the tubule network (A) and treated conditions were compared by number of counted loops (loops determined to be completely closed structures) (B). Data points show number of loops per field as percent of control‐treated cells and are a mean of five independent experiments ± SEM. Treatments consist of LL‐37 (black), CSA‐13 (gray), CSA‐44 (dark blue), CSA‐90 (light blue), CSA‐192 (red), CSA‐131 (pink), CSA‐138 (dark green), and CSA‐142 (light green). The levels of statistical significance of the treatment compared to the media control are as follows: **P < 0.01; *P < 0.05 (two‐way ANOVA with Bonferroni post‐test). ZM 323881 at 10 mmol·L^−1^ reduced the ability of 1 μg·mL^−1^ CSA‐13 to stimulate tube formation (C). Data points show number of loops per field as percent of control‐treated cells and are a mean of six independent experiments ± SEM. The level of statistical significance of the treatment compared to the media control is as follows: *P < 0.05 (ANOVA with Dunnett's post‐test).

Tube formation stimulated with LL‐37 and ceragenins in HUVEC culture on matrigel was used to assess impacts on angiogenesis, an important wound‐healing process. Full growth media (positive control) showed an intact network of HUVEC loops. Testing of LL‐37 and ceragenins was performed on a background of basal EBM + 2% FBS media (Fig. 6A). Compared to the 2% FBS control, LL‐37 treatment led to significantly more loops at all three concentrations tested (0.1, 1, and 10 μg·mL^−1^). Similarly, CSA‐13 and CSA‐90 significantly increased the number of loops at 0.1 and 1 μg·mL^−1^ and CSA‐142 significantly increased the number of loops at 10 μg·mL^−1^ (Fig. 6B). Because VEGFR plays a central role in angiogenesis, we determined if it plays a role in CSA‐13‐induced tube formation. When 10 μmol·L^−1^ ZM 323881 (VEGFR2 inhibitor) was added to 1 μg·mL^−1^ CSA‐13, the formation of the HUVEC tubal network was reduced to the level similar to that of the unstimulated control (Fig. 6C). This result suggests that CSA‐13 may act through the VEGFR2 receptor when stimulating tube formation. ZM 323881 did not have a significant impact on other ceragenins tested and, when used with control media‐treated cells, there was no significant decrease in the amount of loops (data not shown).

### CSA‐13 induced calcium response

**Figure 7:**
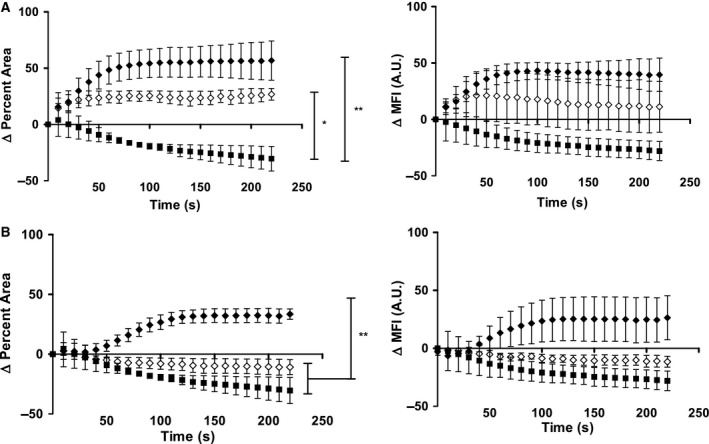
Change in percent area fluo‐4 positive cells and mean fluorescence intensity (MFI) when treated with CSA‐13. (A) Treatment consists of CSA‐13 at 5 μg·mL^−1^ (closed diamonds), CSA‐13 pretreated with inhibitor 10 μmol·L^−1^ ZM 323881 (open diamonds), and control (closed squares). Change in percent area fluo‐4 positive cells and mean fluorescence intensity (MFI) when treated with VEGF. (B) Treatment consists of VEGF at 50 ng·mL^−1^ (closed diamonds), VEGF pretreated with 10 μmol·L^−1^ inhibitor ZM 323881 (open diamonds) and control (closed squares). Data points track change in percent area and MFI over time normalized to positive control ionomycin (100%). Data points are a mean of three independent experiments ± SEM. Area under the curve (AUC) for each group was calculated with graphpad prism, and the levels of statistical significance are as follows: **P < 0.01; *P < 0.05 (ANOVA with Tukey's post‐test).

Intracellular calcium is a secondary messenger in the VEGFR2 pathway. Calcium release in HUVEC can be observed via a fluorescent probe, which allowed determination of induction from either the endoplasmic reticulum intracellularly or the extracellular media. Notably, both CSA‐13 and VEGF induced an increase in fluorescence of HUVEC intracellularly. To quantify these differences, the change in percent fluorescent area and mean fluorescence intensity (MFI) was normalized to the positive control, ionomycin. The area under the curve (AUC) was calculated for the data. Both CSA‐13 and CSA‐13 with inhibitor ZM 323881 significantly increased the AUC compared with the control for the change in percent area. There was a knock down of the effect with ZM present as compared with CSA‐13 alone, but this difference was not significant. These trends were similar for the change in MFI (Fig. 7A). For VEGF (Fig. 7B), ZM significantly decreased the change in percent area AUC down and back to baseline. These trends were similar for the change in MFI.

## Discussion

Previously, CSA‐13 has been shown to impair biofilm formation and viability with mono‐species Gram‐positive and Gram‐negative organisms 17, 28. Our study is the first of its kind to show that CSA‐13 and several other ceragenins can inhibit bacterial cell viability and matrix production in an *in vitro* mixed‐species biofilm consisting of *P. aeruginosa* and *S. aureus*. In addition, several ceragenins affected the size and distribution of bacterial mean cell diameters. While having a marked effect on biofilms, ceragenins also impacted cells important to wound healing, such as keratinocytes and endothelial cells. The HaCaT cell line cannot tolerate high amounts of ceragenins, with toxicity starting at a dosage of 10 μg·mL^−1^ for most ceragenins; at lower doses, however, HaCaT migration was induced by CSA‐13 and CSA‐192 in the ECIS system. HUVEC tube formation was induced by CSA‐13, 90, and 142. For CSA‐13 specifically, HUVEC tube formation and intracellular calcium release were inhibited by ZM 323881, a selective inhibitor for VEGFR2, suggesting that one signaling mechanism activated is the VEGFR2 pathway.

Over the past 15 years, ceragenins have shown promise as alternative antimicrobial treatments, effectively inhibiting and killing of numerous species of bacteria, including drug‐resistant organisms 16, 29. Our results are consistent with these findings, as many of the ceragenins tested had MICs <10 μg·mL^−1^. Also, out of the seven down‐selected ceragenins, all but one (CSA‐44) performed significantly better than peptide LL‐37 at decreasing the amount of viable cells within the mixed‐species biofilm. The mechanism of action for ceragenins has been studied previously: ceragenin selectively associate with bacterial membrane, causing defects which lead to membrane depolarization and cell death 30. Ceragenins related to CSA‐13 with a longer lipid chain at C24, for example CSA‐131, have shown improved potency over CSA‐13 against colistin‐resistant Gram‐negative bacteria 29. Another attractive feature of using ceragenins is their documented synergy with AMPs and antibiotics 31, 32.

Scanning electron microscopy confirmed that *S. aureus* was the predominant species of the target mixed‐species biofilms in our study. *Pseudomonas*, however, can be detected in these biofilms. The SEM images also revealed that for many ceragenins, there are regions of the treated biofilm devoid of cells and/or matrix. Upon quantification, CSA‐13 and CSA‐90 caused a significant drop in matrix levels. This is in contrast to what has been shown previously for CSA‐13 17: when PAO1 biofilms were treated for 25 min, there was a significant killing without any disruption of the biofilm extracellular structure. However, in our current model, the incubation time with the drug was much longer (20 h). Although active CSA‐13 likely does not persist the entire incubation time, the observed killing effect (>99% reduction) is likely significant enough such that there would not be enough cells remaining to produce additional extracellular material. Alternatively, assault on the biofilm by ceragenins may affect the stress response of the bacteria, causing a dysregulation of matrix production‐associated genes. At present, it is still unclear about the mechanisms by which some of the ceragenins could cause reduction in matrix production because in some cases (e.g., CSA‐192 and CSA‐131), reductions of viable biofilm cells did not result in significant decreases of matrix substances in treated biofilms.

Changes in cell size were also observed in our treated biofilm cells as revealed by the SEM images. With CSA‐131, the average cell diameter increased, with some cells having a diameter double the control media‐treated cells. The size change appeared for our *S. aureus* dominated mixed‐species biofilm, but not in preliminary tests with *P. aeruginosa* mono‐species biofilm alone. Prior work has shown that some antimicrobial agents can cause deficiencies in cell division, where the cell accumulates genetic material and forms septa, but never divides, leading to increased cell diameters 33, 34. Alternatively, with CSA‐13 and CSA‐44 treatment, the average cell diameter decreased, with some cells having a diameter half the control media‐treated cells. It is unknown whether these smaller cells are still viable or are simply represent bacterial‐derived vesicles.

Ceragenins exhibited toxicity toward HaCaT cells at low concentrations in a high throughput 96‐well format. HaCaT cells are immortalized human keratinocytes, which makes them a useful model for testing effects on keratinocyte function 35. However, there are limitations to this cell line, as there are membrane differences 36 that can impact how AMPs and ceragenins interact with the cells, and prior studies have shown that they can overestimate the toxicity of a compound compared to what is observed *in vivo* 37. In addition, tissue culture is a simplified cellular environment. Lung epithelial cells that secrete mucin can withstand up to 30 μg·mL^−1^ CSA‐13 without detrimental effects 31. This suggests that, in a more complex physiological environment, matrix molecules can interact with ceragenins and lessen the effect on mammalian cells. Delivery systems (Pluronic F127 surfactant, magnetic nanoparticles) have also been explored to improve interactions with host tissue while retaining antibacterial potency 38.

Through the ECIS system, we show that low concentrations of CSA‐13 and CSA‐192 significantly stimulated HaCaT migration. The purpose of the ECIS system is to screen for possible inducers of migration in a high throughput manner since the system utilizes 96‐well plates. We observed well‐to‐well variability within a plate and plate‐to‐plate variability. This effect was independent of cell variability between experiments. Also, the cell‐covered area on the electrode is 0.256 mm^2^, which at least 100× smaller than even the smallest wounds used in excisional mouse models. Nonetheless, we did see a reproducible effect with CSA‐13 and CSA‐192 which encourages further investigation using clinically relevant wound‐healing models. Preliminary investigations into HaCaT gene expression changes when stimulated at ECIS concentration levels did not show significant differences in EGF, VEGF, matrix metalloproteinase‐1, and inflammatory cytokines IL‐8 and IL‐1α (data not shown). Previous work has suggested the pleiotropy of CSA‐13: in a murine skin model of vaccinia virus infection, treatment with CSA‐13 decreased the amount of satellite lesions. CSA‐13 was also able to upregulate LL‐37 and human β‐Defensin‐3 gene expression in cultures of primary human keratinocytes 39. It is, therefore, possible that ceragenins acting on cells can induce a desirable function (i.e. migration) and upregulate genes that also induce the same function.

Along with migration, *in vitro* tube formation was induced with CSA‐13, 90, and 142. Tube formation is one of the preferred methods for testing an angiogenesis response to a single factor because it is short‐term and reproducible. Because this assay is 2‐D, it does not entirely recapitulate *in vivo* architecture 40. We showed that CSA‐13‐induced tube formation is inhibited by the VEGFR2 inhibitor ZM 323881, suggesting that CSA‐13 may signal through this receptor. In VEGFR2 signaling, activation of the tyrosine kinase receptor leads to the phosphorylation of phospholipase C that causes the release of Ca^2+^, the secondary messenger, into the cytoplasm from the endoplasmic reticulum and extracellular environment. Ca^2+^ release propagates signals that lead to cell proliferation, migration, and tubulogenesis, and it is, therefore, a good measure of pathway activation. We measured an increase in intracellular calcium with VEGF, CSA‐13, and ionomycin (positive control) treatment. When pre‐incubated with the inhibitor, we saw that VEGF Ca^2+^ signaling was completely inhibited, while CSA‐13 Ca^2+^ signaling was only partially inhibited. This suggests that CSA‐13 can also target other pathways where Ca^2+^ signaling occurs. Concentrations of VEGF used to induce Ca^2+^ signaling (~50 ng·mL^−1^) are higher than physiological levels but typical of other studies using this assay 41.

We have shown that the ceragenin class of molecules has a profound effect on mixed‐species biofilms, including cell viability, cell morphology, and matrix production. These findings are in addition to the growing literature showing ceragenins as stable, potent antimicrobials. In addition, ceragenins have the ability to interact with mammalian cells, stimulating migration and tube formation *in vitro*. Among the ceragenins, CSA‐13 had the most significant impact on biofilms and mammalian cells. While further *in vivo* testing in animal wounding models is necessary to confirm their pleiotropic activity, ceragenins have the potential to not only be topical antimicrobials, but also wound adjunct therapies.

## Author contributions

MAO, TY, PBS, and KPL conceived and designed the project. MAO and TY acquired the data. MAO, TY, PBS, and KPL analyzed and interpreted the data. MO and KPL wrote the article.
